# Tetra-μ-acetato-κ^8^ *O*:*O*′-bis­[(3-chloro­pyridine-κ*N*)ruthenium(II,III)](*Ru*—*Ru*) hexa­fluorido­phosphate 1,2-di­chloro­ethane monosolvate

**DOI:** 10.1107/S2414314622002498

**Published:** 2022-03-10

**Authors:** Anthony J. Aquino, Daniel Gerrior, T. Stanley Cameron, Katherine N. Robertson, Manuel A.S. Aquino

**Affiliations:** a Department of Chemistry, St. Francis Xavier University, PO Box 5000, Antigonish, NS, Canada, B2G 2W5; b Department of Chemistry, Dalhousie University, 6274 Coburg Rd, Halifax, NS, Canada, B3H 4R2; c Department of Chemistry, Saint Mary’s University, Halifax, NS, Canada, B3H 3C3; Vienna University of Technology, Austria

**Keywords:** crystal structure, coordination compound, mixed-valency, diruthenium tetra­carboxyl­ate

## Abstract

The title compound, [Ru_2_(μ-O_2_CCH_3_)_4_(C_5_H_4_ClN)_2_]PF_6_·C_2_H_4_Cl_2_, was obtained *via* a rapid substitution reaction of 3-chloro­pyridine for water in [Ru_2_(μ-O_2_CCH_3_)_4_(H_2_O)_2_]PF_6_ in 2-propanol and subsequent crystallization from a di­chloro­ethane solution. The cationic diruthenium(II,III) tetra­acetate core lies on a crystallographic inversion center with Ru—Ru and Ru—N bond lengths of 2.2738 (3) and 2.2920 (17) Å, respectively. The Ru—Ru—N bond angle is close to linear at 176.48 (4)°, and a significant π-stacking inter­action of 3.5649 (16) Å is seen between overlapping pyridine rings of adjacent cations.

## Abstract 2

The mixed-valent cationic complex of the solvated title salt, [Ru_2_(μ-O_2_CCH_3_)_4_(C_5_H_4_ClN)_2_]PF_6_·C_2_H_4_Cl_2_, exhibits a classic paddlewheel or lantern structure with each Ru atom in a slightly distorted octa­hedral environment.

## Graphical abstract

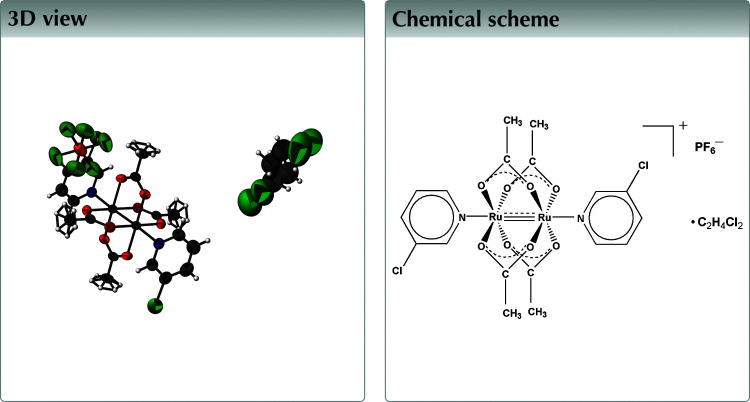

## Structure description

Earlier research in our lab dealt with the chemistry of various mixed-valent diruth­enium(II,III) tetra­acetate complexes incorporating substituted pyridines and other, biologically relevant, heterocyclic N-donors in the axial coordination positions (Bland *et al.*, 2005 ▸; Gilfoy *et al.*, 2001 ▸; Minaker *et al.*, 2011 ▸; Vamvounis *et al.*, 2000 ▸). At that time we were unable to obtain structures of amino- or chloro-pyridine diadducts. Recently, we have been able to characterize both a 3-amino­pyridine diadduct (Aquino *et al.*, 2021 ▸) and the 3-chloro­pyridine diadduct is reported here. This is the first crystal structure of a chloro-pyridine diadduct of a diruthenium(II,III) tetra­carboxyl­ate that we are aware of.

**Figure 1:**
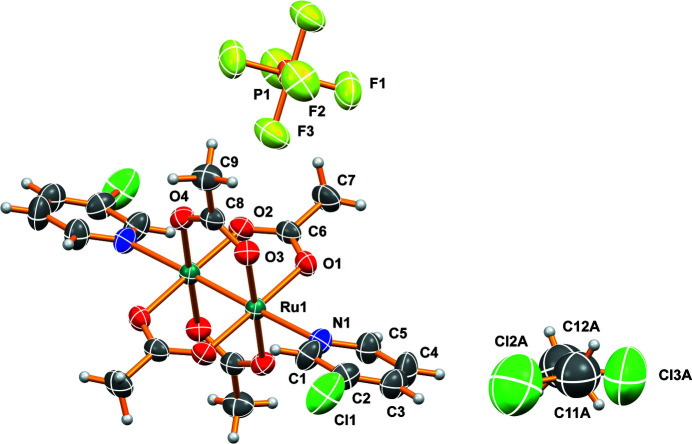
The mol­ecular structure of the title compound with displacement ellipsoids at the 50% probability level. Unlabeled atoms are generated by the symmetry operations (i) (−*x* + 1, −*y*, −*z*) and (ii) (−*x* + 2, −*y*, −*z* + 1). Only one orientation of the disordered methyl groups and the disordered C_2_H_4_Cl_2_ solvent mol­ecule is shown

**Figure 2:**
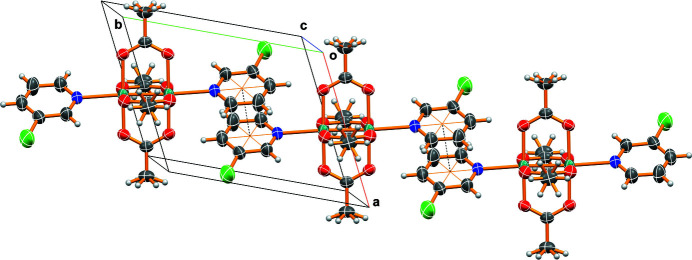
Packing diagram viewed approximately along [001] showing the π–π stacking inter­actions (dashed lines).

The solvated title salt consists of a complex cation with a diruthenium (II,III) tetra­acetate core and 3-chloro­pyridines in the axial positions, a hexa­fluorido­phophate anion, and a 1,2-di­chloro­ethane mol­ecule of solvation (Fig. 1 ▸). The cation displays the classic Chinese lantern or paddlewheel shape with each ruthenium atom at the center of a slightly distorted octa­hedron. The Ru1—Ru1(−*x* + 1, −*y*, −*z*) and Ru1—N1 bond lengths are 2.2738 (3) and 2.2920 (17) Å, and are similar to those in the 3-cyano­pyridine diadduct [2.2702 (6) and 2.295 (3) Å; Minaker *et al.*, 2011 ▸]. The Ru1(−*x* + 1, −*y*, −*z*)—Ru1—N1 bond angle of 176.48 (4)° is also comparable to the 174.27 (7)° of the 3-cyano­pyridine adduct, showing essentially linear coordination. While no substantial hydrogen bonding was detected in the title compound, a significant π–π stacking inter­action between pyridine rings of adjacent complexes was noted (Fig. 2 ▸) and creates a chain motif along [010]. The distance between the ring centroids (N1, C1–C5) is 3.5649 (16) Å with a slippage of 0.553 Å, the symmetry code to generate the second ring being (1 − *x*, 1 − *y*, −*z*).

## Synthesis and crystallization

Synthesis of the title compound followed an earlier method developed in our lab (Vamvounis *et al.*, 2000 ▸). [Ru_2_(μ-O_2_CCH_3_)_4_(H_2_O)_2_]PF_6_ (0.100 g, 0.161 mmol) was dissolved in 10 ml of 2-propanol. Then, 3-chloro­pyridine (0.0732 g, 0.645 mmol) was added and the solution allowed to stir for 5 min at room temperature. The volume of the solution was then reduced to 5 ml under vacuum and allowed to cool to 278 K overnight. The crystalline product was collected *via* suction filtration. Yield = 0.098 g (63%). Crystals suitable for X-ray diffraction were obtained by slow diffusion of diethyl ether into a 1,2-di­chloro­ethane solution of the complex. IR (cm^−1^): 2947 (νC—H), 1447 (*asym*. νCOO), 1396 (*sym*. νCOO), 841, (νPF_6_), 766 (νC—Cl), 692 (δC—CH_3_). UV–vis (λ nm, (log ɛ)): 427 (2.95), 263 (4.05), 210 (4.33).

## Refinement

Crystal data, data collection and structure refinement details are summarized in Table 1 ▸. Two reflections were removed from the refinement because of poor agreements between *F* ^2^(obs) and *F* ^2^(calc), 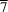 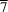 5 and 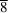 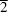 6. In the cation, the methyl groups of the acetate ligands were modeled in the refinement as idealized disordered methyl groups with the two sets of positions rotated from each other by 60°. The crystal structure was found to contain solvent mol­ecules. The recrystallization solvents were di­chloro­ethane and diethyl ether. The SQUEEZE routine (Spek, 2015 ▸) in *PLATON* (Spek, 2020 ▸) was used to get an estimate of the void volumes and of the unaccounted electron density in them. The unit cell was found to contain one void of 228 Å^3^ with 50 electrons per void. This suggested that there was one mol­ecule of di­chloro­ethane in each void and it was modeled as such. The disorder in the solvent was modeled by two equally occupied parts, which were then also split again across an inversion center, giving all atoms an occupancy of 0.25. The geometries of all the parts were restrained to be similar. In addition the C—C and the C—Cl bond lengths were restrained to reasonable values. The heavy atoms of the same type in the solvent were restrained to have similar displacement parameters and the carbon atoms were restrained to have more isotropic ellipsoids. Finally, rigid-bond restraints were placed over each solvent part.

## Supplementary Material

Crystal structure: contains datablock(s) I. DOI: 10.1107/S2414314622002498/wm4161sup1.cif

Structure factors: contains datablock(s) I. DOI: 10.1107/S2414314622002498/wm4161Isup2.hkl

CCDC reference: 2156199

Additional supporting information:  crystallographic information; 3D view; checkCIF report

## Figures and Tables

**Table 1:** Experimental details

Crystal data	
Chemical formula	[Ru_2_(C_2_H_3_O_2_)_4_(C_5_H_4_ClN)_2_]PF_6_
*M*_r_	909.32
Crystal system, space group	Triclinic, *P* 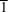
Temperature (K)	293
*a*, *b*, *c* (Å)	8.2737 (1), 10.5784 (3), 11.5534 (1)
α, β, γ (°)	100.764 (7), 108.980 (8), 110.525 (7)
*V* (Å^3^)	842.27 (6)
*Z*	1
Radiation type	Mo *K*α
μ (mm^−1^)	1.34
Crystal size (mm)	0.43 × 0.20 × 0.07

Data collection	
Diffractometer	Rigaku R-AXIS RAPID
Absorption correction	Multi-scan (*ABSCOR*; Higashi, 1995 ▸).
*T*_min_, *T*_max_	0.702, 0.921
No. of measured, independent and observed [*I* > 2σ(*I*)] reflections	23200, 4084, 4084
*R*_int_	0.084
(sin θ/λ)_max_ (Å^−1^)	0.687

Refinement	
*R*[*F* ^2^ > 2σ(*F* ^2^)], *wR*(*F* ^2^), *S*	0.024, 0.069, 1.10
No. of reflections	4084
No. of parameters	250
No. of restraints	99
H-atom treatment	H-atom parameters constrained
Δρ_max_, Δρ_min_ (e Å^−3^)	0.49, −0.52

Computer programs: *CrystalStructure* (Rigaku, 2007 ▸), *SIR2004* (Burla *et al.*, 2005 ▸), *SHELXL* (Sheldrick, 2015 ▸), Merdury (Macrae *et al.*, 2020 ▸) and *publCIF* (Westrip, 2010 ▸).

## full crystallographic data

### Crystal data

**Table:**

[Ru_2_(C_2_H_3_O_2_)_4_(C_5_H_4_ClN)_2_]PF_6_	*Z* = 1
*M**_r_* = 909.32	*F*(000) = 447
Triclinic, *P*1	*D*_x_ = 1.793 Mg m^−^^3^
*a* = 8.2737 (1) Å	Mo *K*α radiation, λ = 0.71075 Å
*b* = 10.5784 (3) Å	Cell parameters from 8636 reflections
*c* = 11.5534 (1) Å	θ = 2.7–58.1°
α = 100.764 (7)°	µ = 1.34 mm^−^^1^
β = 108.980 (8)°	*T* = 293 K
γ = 110.525 (7)°	Needle plate, light brown
*V* = 842.27 (6) Å^3^	0.43 × 0.20 × 0.07 mm

### Data collection

**Table:**

Rigaku R-AXIS RAPID diffractometer	4084 reflections with *I* > 2σ(*I*)
Detector resolution: 10.00 pixels mm^-1^	*R*_int_ = 0.084
ω scans	θ_max_ = 29.2°
Absorption correction: multi-scan (*ABSCOR*; Higashi, 1995).	*h* = −11→11
*T*_min_ = 0.702, *T*_max_ = 0.921	*k* = −14→14
23200 measured reflections	*l* = −15→15
4084 independent reflections	

### Refinement

**Table:**

Refinement on *F*^2^	Primary atom site location: structure-invariant direct methods
Least-squares matrix: full	Secondary atom site location: iterative
*R*[*F*^2^ > 2σ(*F*^2^)] = 0.024	Hydrogen site location: inferred from neighbouring sites
*wR*(*F*^2^) = 0.069	H-atom parameters constrained
*S* = 1.10	*w* = 1/[σ^2^(*F*_o_^2^) + (0.0183*P*)^2^] where *P* = (*F*_o_^2^ + 2*F*_c_^2^)/3
4084 reflections	(Δ/σ)_max_ = 0.002
250 parameters	Δρ_max_ = 0.49 e Å^−^^3^
99 restraints	Δρ_min_ = −0.51 e Å^−^^3^

### Special details

**Table:**

Geometry. All esds (except the esd in the dihedral angle between two l.s. planes) are estimated using the full covariance matrix. The cell esds are taken into account individually in the estimation of esds in distances, angles and torsion angles; correlations between esds in cell parameters are only used when they are defined by crystal symmetry. An approximate (isotropic) treatment of cell esds is used for estimating esds involving l.s. planes.

### Fractional atomic coordinates and isotropic or equivalent isotropic displacement parameters (Å^2^)

**Table:**

	*x*	*y*	*z*	*U*_iso_*/*U*_eq_	Occ. (<1)
Ru1	0.53229 (2)	0.11769 (2)	0.03458 (2)	0.03317 (6)	
Cl1	0.94986 (12)	0.69082 (8)	0.05336 (11)	0.0947 (3)	
P1	1.000000	0.000000	0.500000	0.0569 (2)	
F1	0.9249 (3)	0.0893 (2)	0.57695 (18)	0.0917 (6)	
F2	0.8293 (3)	−0.1428 (2)	0.4802 (2)	0.0949 (6)	
F3	0.8683 (2)	0.0034 (2)	0.36556 (16)	0.0862 (5)	
O1	0.5024 (2)	0.09675 (16)	0.19771 (13)	0.0425 (3)	
O2	0.5603 (2)	0.13381 (16)	−0.13033 (14)	0.0416 (3)	
O3	0.81269 (19)	0.16819 (15)	0.12454 (14)	0.0415 (3)	
O4	0.25099 (19)	0.06299 (16)	−0.05668 (14)	0.0410 (3)	
N1	0.6166 (2)	0.35849 (18)	0.10681 (18)	0.0427 (4)	
C1	0.7380 (3)	0.4444 (2)	0.0712 (3)	0.0544 (5)	
H1	0.787140	0.404761	0.020620	0.065*	
C2	0.7934 (3)	0.5901 (2)	0.1069 (2)	0.0541 (5)	
C3	0.7242 (4)	0.6514 (2)	0.1813 (3)	0.0615 (6)	
H3	0.759615	0.749444	0.205596	0.074*	
C4	0.6005 (4)	0.5631 (3)	0.2192 (3)	0.0692 (7)	
H4	0.550116	0.600878	0.269981	0.083*	
C5	0.5508 (4)	0.4172 (3)	0.1814 (2)	0.0564 (5)	
H5	0.468896	0.358717	0.209052	0.068*	
C6	0.4646 (3)	−0.0242 (2)	0.21398 (18)	0.0417 (4)	
C7	0.4480 (4)	−0.0360 (3)	0.3375 (2)	0.0600 (6)	
H7A	0.470433	0.055322	0.390462	0.090*	0.5
H7B	0.322347	−0.106306	0.316927	0.090*	0.5
H7C	0.540552	−0.064512	0.384004	0.090*	0.5
H7D	0.418455	−0.132319	0.337133	0.090*	0.5
H7E	0.566541	0.029309	0.410669	0.090*	0.5
H7F	0.348336	−0.012486	0.343592	0.090*	0.5
C8	0.1350 (3)	−0.0685 (2)	−0.11919 (18)	0.0402 (4)	
C9	−0.0728 (3)	−0.1078 (3)	−0.1872 (2)	0.0562 (5)	
H9A	−0.143002	−0.209827	−0.230077	0.084*	0.5
H9B	−0.116969	−0.078012	−0.124702	0.084*	0.5
H9C	−0.091531	−0.060687	−0.250386	0.084*	0.5
H9D	−0.091332	−0.022523	−0.173366	0.084*	0.5
H9E	−0.117366	−0.154338	−0.278742	0.084*	0.5
H9F	−0.142804	−0.171664	−0.153057	0.084*	0.5
Cl2A	0.218 (3)	0.548 (2)	0.3742 (19)	0.233 (6)	0.25
C11A	0.138 (5)	0.586 (4)	0.499 (3)	0.162 (8)	0.25
H11A	0.241338	0.623432	0.584600	0.194*	0.25
H11B	0.086968	0.655324	0.488739	0.194*	0.25
C12A	−0.014 (7)	0.444 (3)	0.477 (5)	0.154 (7)	0.25
H12A	0.038817	0.383465	0.511063	0.184*	0.25
H12B	−0.101345	0.394270	0.385840	0.184*	0.25
Cl3A	−0.126 (4)	0.506 (3)	0.571 (2)	0.290 (10)	0.25
Cl2B	0.202 (3)	0.5888 (19)	0.454 (3)	0.212 (6)	0.25
C11B	0.035 (6)	0.589 (4)	0.525 (5)	0.155 (7)	0.25
H11C	0.102125	0.668383	0.607326	0.186*	0.25
H11D	−0.064456	0.605649	0.467743	0.186*	0.25
C12B	−0.053 (4)	0.455 (4)	0.549 (4)	0.160 (7)	0.25
H12C	0.025345	0.456765	0.633197	0.192*	0.25
H12D	−0.075663	0.372165	0.481778	0.192*	0.25
Cl3B	−0.275 (3)	0.452 (3)	0.544 (2)	0.254 (9)	0.25

### Atomic displacement parameters (Å^2^)

**Table:**

	*U*^11^	*U*^22^	*U*^33^	*U*^12^	*U*^13^	*U*^23^
Ru1	0.03692 (9)	0.03044 (9)	0.03524 (9)	0.01429 (7)	0.01879 (7)	0.01260 (7)
Cl1	0.0931 (5)	0.0526 (4)	0.1638 (9)	0.0269 (4)	0.0820 (6)	0.0510 (5)
P1	0.0473 (4)	0.0698 (5)	0.0440 (4)	0.0201 (4)	0.0198 (3)	0.0101 (4)
F1	0.0812 (12)	0.1152 (16)	0.0748 (11)	0.0509 (11)	0.0357 (9)	0.0047 (10)
F2	0.0731 (11)	0.0886 (13)	0.0909 (14)	0.0053 (10)	0.0353 (10)	0.0225 (11)
F3	0.0722 (10)	0.1275 (16)	0.0568 (9)	0.0446 (11)	0.0237 (8)	0.0329 (10)
O1	0.0500 (8)	0.0477 (8)	0.0356 (7)	0.0228 (6)	0.0237 (6)	0.0144 (6)
O2	0.0478 (7)	0.0434 (7)	0.0423 (7)	0.0192 (6)	0.0257 (6)	0.0234 (6)
O3	0.0348 (6)	0.0399 (7)	0.0453 (7)	0.0123 (5)	0.0165 (6)	0.0142 (6)
O4	0.0402 (7)	0.0439 (7)	0.0471 (8)	0.0224 (6)	0.0220 (6)	0.0190 (6)
N1	0.0444 (9)	0.0320 (8)	0.0489 (9)	0.0155 (7)	0.0197 (7)	0.0111 (7)
C1	0.0560 (12)	0.0392 (10)	0.0764 (15)	0.0215 (9)	0.0368 (11)	0.0211 (10)
C2	0.0475 (11)	0.0378 (10)	0.0696 (14)	0.0139 (8)	0.0207 (10)	0.0197 (10)
C3	0.0679 (15)	0.0365 (10)	0.0662 (15)	0.0200 (10)	0.0193 (12)	0.0108 (10)
C4	0.0924 (19)	0.0481 (13)	0.0720 (17)	0.0325 (13)	0.0450 (15)	0.0100 (12)
C5	0.0654 (14)	0.0451 (11)	0.0606 (13)	0.0210 (10)	0.0344 (11)	0.0150 (10)
C6	0.0390 (9)	0.0567 (11)	0.0375 (9)	0.0219 (8)	0.0214 (7)	0.0220 (8)
C7	0.0685 (14)	0.0859 (18)	0.0451 (11)	0.0377 (13)	0.0361 (11)	0.0348 (12)
C8	0.0371 (8)	0.0506 (10)	0.0391 (9)	0.0195 (8)	0.0206 (7)	0.0201 (8)
C9	0.0382 (10)	0.0688 (15)	0.0622 (13)	0.0222 (10)	0.0211 (9)	0.0270 (11)
Cl2A	0.219 (10)	0.219 (11)	0.261 (16)	0.095 (8)	0.117 (11)	0.061 (12)
C11A	0.159 (9)	0.158 (9)	0.161 (9)	0.072 (7)	0.059 (6)	0.051 (7)
C12A	0.154 (8)	0.153 (9)	0.156 (8)	0.073 (7)	0.061 (6)	0.056 (7)
Cl3A	0.38 (2)	0.29 (2)	0.204 (12)	0.23 (2)	0.084 (16)	0.008 (13)
Cl2B	0.245 (12)	0.171 (9)	0.239 (16)	0.097 (8)	0.135 (10)	0.043 (10)
C11B	0.156 (8)	0.153 (8)	0.158 (9)	0.075 (7)	0.060 (6)	0.053 (6)
C12B	0.156 (9)	0.157 (9)	0.161 (9)	0.076 (7)	0.053 (6)	0.056 (7)
Cl3B	0.34 (2)	0.339 (19)	0.128 (8)	0.22 (2)	0.080 (12)	0.071 (10)

### Geometric parameters (Å, º)

**Table:**

Ru1—O1	2.0204 (14)	C7—H7A	0.9600
Ru1—O2	2.0232 (14)	C7—H7B	0.9600
Ru1—O4	2.0235 (13)	C7—H7C	0.9600
Ru1—O3	2.0256 (13)	C7—H7D	0.9600
Ru1—Ru1^i^	2.2738 (3)	C7—H7E	0.9600
Ru1—N1	2.2920 (17)	C7—H7F	0.9600
Cl1—C2	1.730 (3)	C8—C9	1.498 (3)
P1—F2^ii^	1.5795 (19)	C9—H9A	0.9600
P1—F2	1.5795 (19)	C9—H9B	0.9600
P1—F1^ii^	1.5896 (18)	C9—H9C	0.9600
P1—F1	1.5896 (18)	C9—H9D	0.9600
P1—F3^ii^	1.5965 (16)	C9—H9E	0.9600
P1—F3	1.5965 (16)	C9—H9F	0.9600
O1—C6	1.272 (2)	Cl2A—C11A	1.803 (16)
O2—C6^i^	1.267 (3)	C11A—C12A	1.493 (13)
O3—C8^i^	1.272 (2)	C11A—H11A	0.9700
O4—C8	1.271 (2)	C11A—H11B	0.9700
N1—C5	1.329 (3)	C12A—Cl3A	1.811 (16)
N1—C1	1.331 (3)	C12A—H12A	0.9700
C1—C2	1.379 (3)	C12A—H12B	0.9700
C1—H1	0.9300	Cl2B—C11B	1.826 (16)
C2—C3	1.364 (4)	C11B—C12B	1.476 (13)
C3—C4	1.373 (4)	C11B—H11C	0.9700
C3—H3	0.9300	C11B—H11D	0.9700
C4—C5	1.389 (3)	C12B—Cl3B	1.805 (16)
C4—H4	0.9300	C12B—H12C	0.9700
C5—H5	0.9300	C12B—H12D	0.9700
C6—C7	1.501 (3)		
			
O1—Ru1—O2	178.70 (5)	N1—C5—H5	119.0
O1—Ru1—O4	90.18 (6)	C4—C5—H5	119.0
O2—Ru1—O4	89.69 (6)	O2^i^—C6—O1	122.70 (17)
O1—Ru1—O3	89.85 (6)	O2^i^—C6—C7	119.34 (19)
O2—Ru1—O3	90.25 (6)	O1—C6—C7	117.96 (19)
O4—Ru1—O3	178.83 (5)	C6—C7—H7A	109.5
O1—Ru1—Ru1^i^	89.66 (4)	C6—C7—H7B	109.5
O2—Ru1—Ru1^i^	89.04 (4)	H7A—C7—H7B	109.5
O4—Ru1—Ru1^i^	89.73 (4)	C6—C7—H7C	109.5
O3—Ru1—Ru1^i^	89.11 (4)	H7A—C7—H7C	109.5
O1—Ru1—N1	91.38 (6)	H7B—C7—H7C	109.5
O2—Ru1—N1	89.92 (6)	H7D—C7—H7E	109.5
O4—Ru1—N1	93.63 (6)	H7D—C7—H7F	109.5
O3—Ru1—N1	87.53 (6)	H7E—C7—H7F	109.5
Ru1^i^—Ru1—N1	176.48 (4)	O4—C8—O3^i^	122.84 (17)
F2^ii^—P1—F2	180.0	O4—C8—C9	118.51 (18)
F2^ii^—P1—F1^ii^	89.08 (12)	O3^i^—C8—C9	118.64 (18)
F2—P1—F1^ii^	90.92 (12)	C8—C9—H9A	109.5
F2^ii^—P1—F1	90.92 (12)	C8—C9—H9B	109.5
F2—P1—F1	89.08 (12)	H9A—C9—H9B	109.5
F1^ii^—P1—F1	180.00 (15)	C8—C9—H9C	109.5
F2^ii^—P1—F3^ii^	88.89 (11)	H9A—C9—H9C	109.5
F2—P1—F3^ii^	91.11 (11)	H9B—C9—H9C	109.5
F1^ii^—P1—F3^ii^	90.71 (11)	H9D—C9—H9E	109.5
F1—P1—F3^ii^	89.29 (11)	H9D—C9—H9F	109.5
F2^ii^—P1—F3	91.11 (11)	H9E—C9—H9F	109.5
F2—P1—F3	88.89 (11)	C12A—C11A—Cl2A	104.0 (18)
F1^ii^—P1—F3	89.29 (11)	C12A—C11A—H11A	111.0
F1—P1—F3	90.71 (11)	Cl2A—C11A—H11A	110.9
F3^ii^—P1—F3	180.0	C12A—C11A—H11B	111.0
C6—O1—Ru1	118.99 (13)	Cl2A—C11A—H11B	111.0
C6^i^—O2—Ru1	119.58 (12)	H11A—C11A—H11B	109.0
C8^i^—O3—Ru1	119.40 (12)	C11A—C12A—Cl3A	99.1 (17)
C8—O4—Ru1	118.90 (12)	C11A—C12A—H12A	112.0
C5—N1—C1	118.17 (19)	Cl3A—C12A—H12A	112.0
C5—N1—Ru1	123.95 (15)	C11A—C12A—H12B	112.0
C1—N1—Ru1	117.87 (15)	Cl3A—C12A—H12B	112.0
N1—C1—C2	122.2 (2)	H12A—C12A—H12B	109.6
N1—C1—H1	118.9	C12B—C11B—Cl2B	114 (2)
C2—C1—H1	118.9	C12B—C11B—H11C	108.8
C3—C2—C1	120.3 (2)	Cl2B—C11B—H11C	108.8
C3—C2—Cl1	121.60 (19)	C12B—C11B—H11D	108.8
C1—C2—Cl1	118.1 (2)	Cl2B—C11B—H11D	108.8
C2—C3—C4	117.6 (2)	H11C—C11B—H11D	107.7
C2—C3—H3	121.2	C11B—C12B—Cl3B	102.6 (18)
C4—C3—H3	121.2	C11B—C12B—H12C	111.3
C3—C4—C5	119.7 (2)	Cl3B—C12B—H12C	111.3
C3—C4—H4	120.2	C11B—C12B—H12D	111.3
C5—C4—H4	120.2	Cl3B—C12B—H12D	111.3
N1—C5—C4	122.1 (2)	H12C—C12B—H12D	109.2
			
C5—N1—C1—C2	−1.4 (4)	Ru1—N1—C5—C4	−177.7 (2)
Ru1—N1—C1—C2	178.28 (17)	C3—C4—C5—N1	−1.3 (4)
N1—C1—C2—C3	0.2 (4)	Ru1—O1—C6—O2^i^	−1.9 (3)
N1—C1—C2—Cl1	−179.25 (19)	Ru1—O1—C6—C7	178.49 (14)
C1—C2—C3—C4	0.5 (4)	Ru1—O4—C8—O3^i^	1.7 (3)
Cl1—C2—C3—C4	179.9 (2)	Ru1—O4—C8—C9	−179.58 (14)
C2—C3—C4—C5	0.1 (4)	Cl2A—C11A—C12A—Cl3A	−164 (3)
C1—N1—C5—C4	1.9 (4)	Cl2B—C11B—C12B—Cl3B	−154 (3)

Symmetry codes: (i) −*x*+1, −*y*, −*z*; (ii) −*x*+2, −*y*, −*z*+1.
